# Quality of life in Klinefelter patients on testosterone replacement therapy compared to healthy controls: an observational study on the impact of psychological distress, personality traits, and coping strategies

**DOI:** 10.1007/s40618-020-01400-8

**Published:** 2020-08-31

**Authors:** M. Fabrazzo, G. Accardo, I. Abbondandolo, G. Goglia, D. Esposito, G. Sampogna, F. Catapano, D. Giugliano, D. Pasquali

**Affiliations:** 1 Department of Psychiatry, University of Campania ‘Luigi Vanvitelli’, Naples, Italy; 2 Unit of Endocrinology and Metabolic Diseases, Department of Advanced Medical and Surgical Science, University of Campania ‘Luigi Vanvitelli’, Naples, Italy; 3 Department of Internal Medicine and Clinical Nutrition, Institute of Medicine, Sahlgrenska Academy, University of Gothenburg, Gothenburg, Sweden

**Keywords:** Klinefelter syndrome, Genetic disorder, Quality of life, Testosterone replacement therapy, Personality traits, Coping strategies

## Abstract

**Purpose:**

We aimed to verify if 1 year-testosterone-replacement therapy could produce a psychopathological recovery and a satisfactory quality of life in Klinefelter syndrome (KS) patients compared to matched healthy controls. Further, we analyzed personality traits and coping strategies, an issue not yet examined in androgen-treated KS patients. We also enquired whether any of the sociodemographic and psychological variables might predict a patient’s general and sexual life satisfaction.

**Methods:**

The Quality of Life Enjoyment and Satisfaction Questionnaire and the Temperament and Character Inventory-Revised were administered to both 23 KS patients and matched healthy subjects. Psychopathology was investigated by the Symptom Checklist-90-Revised (SCL-90-R) and the Mini-mental State Examination. The COPE Inventory was used to identify cognitive and behavioral strategies to manage disease-related distress.

**Results:**

In testosterone-treated KS patients, when compared with controls, SCL-90-R subscales analysis evidenced high psychological distress, mainly presented as obsessive thoughts, hanger-hostility, phobias, and psychoticism. Self-directedness and self-transcendence, along with the prevalent use of *emotion-focused* coping strategies, outlined the personality of our KS patients. Depression and somatization proved to be predictors of general life dissatisfaction. Depression, anger-hostility, and paranoid ideation, instead, emerged as predictors of sexual life dissatisfaction.

**Conclusion:**

Endocrinologists should cooperate with mental health providers to foster a better outcome of the disease in KS patients.

## Introduction

Klinefelter syndrome (KS) is one of the most common sex chromosomal abnormalities and is characterized by hypergonadotropic hypogonadism (HH) and infertility [1, 2].

Low testosterone levels can affect physical, social, emotional, cognitive, and sexual functioning, key domains that contribute to health-related quality of life (QoL) in hypogonadal KS men [3–7].

Also, cognitive and language deficits due to genetic factors may influence QoL and lead to adaptation and behavioral problems during adolescence [7].

The neuropsychological phenotype in adults affected by KS varies considerably. Impaired measures of verbal skills, high incidence of dyslexia, and social dysfunctions are among the most reported behavioral phenotypes, which may worsen the perceived stigma associated with the disease and complications. Perceived stigma may induce KS patients to adopt coping strategies, through which they become capable of addressing and managing the genetic disease [8].

Recent studies [9, 10] reported that QoL outcomes—wellbeing, body image and self-esteem, mental and general health—are considerably poorer in KS patients compared to those of the general male population. Also, reduced QoL can cause loss of livelihood and problems in sexual life, including a deficit of libido and erectile dysfunctions [4, 11], which may consequently exacerbate the impact of the disease on psychological profile and perception of wellbeing [12].

Low testosterone levels are generally associated with increased morbidity and mortality [13–15]. Early diagnosis and treatment are, therefore, crucial to ensure the wellbeing and prevent disease-related complications.

Treatment of KS aims to decrease symptoms, enhance functionality, and improve the wellbeing of patients and their family environment, as likewise occurs in most chronic disorders. Testosterone replacement therapy, which is the accepted standard of care in KS patients, indeed, produces no effect on infertility caused by the disease, but corrects the androgen deficiency, and provides patients with appropriate virilization (improved libido, sexual activity, nocturnal erections, sexual thoughts, and sexual satisfaction), considerable increased skeletal muscle strength and hematocrit, plus reduced bone resorption [16–18].

Only a few studies have evaluated so far changes of QoL after testosterone treatment [19–21], and a limited number have included either testosterone-treated and untreated KS patients or healthy controls to analyze such an issue [9, 12]. Finally, no study has yet identified the prevailing personality traits and the coping strategies used to manage QoL, disease-related distress, and health complications in testosterone-treated KS patients.

In the present preliminary observational study, we evaluated whether in KS patients under testosterone undecanoate treatment for one year, QoL yielded a level comparable to that of matched healthy controls. In both groups, we analyzed QoL outcomes and disease-related distress, along with personality traits and coping strategies used to manage the impact of the disease.

We finally enquired whether any of the sociodemographic and psychopathological factors, personality traits, and coping strategies could predict a patient’s general and sexual life satisfaction.

## Material and methods

### Subjects

We conducted our study at the Department of Endocrinology in cooperation with the Department of Psychiatry of the University of Campania ‘Luigi Vanvitelli’, Naples, from September 2012 to January 2019. We used a cross-sectional design to assess whether KS patients, after one-year of testosterone replacement therapy, achieved a QoL comparable to that of a healthy control group. Our cohort included 23 healthy subjects, recruited among medical students and clinical staff, and paired by gender, age, and educational level with 23 adult outpatients with a verified KS karyotype (47, XXY), and treated for one year with parenteral testosterone undecanoate. No significant differences emerged between KS and healthy subjects in either age (*p* < 0.974) or education level (*p* < 0.823). Inclusion criteria for both KS patients and healthy controls required that participants were aged 18–60 years and not androgen treated in the preceding year. Exclusion criteria comprised a history of substance use, neurological disease or head injury, hematocrit at 55% or greater, and a sensitivity to testosterone formulation. In addition, hypogonadism was an exclusion criterion for healthy controls.

In line with Corona et al. [15], we defined hypogonadism as a medical problem associated with testosterone deficiency syndrome along with the presence of symptoms (i.e. sexual dysfunction). KS patients usually show a condition of early-onset hypogonadism due to peripheral defects (i.e. peri-pubertal onset), affecting both phenotype and behavior. Furthermore, we used a total plasma level of 12 nmol/L (346 ng/dL) as a threshold for measuring low testosterone, and two consecutive readings of plasma total testosterone values to diagnose hypogonadism, following the Endocrine Society Guidelines [22] and mostly shared consensus.

In our cohort KS patients, we administered the androgen replacement therapy to our cohort KS patients according to the recommendations of the Italian Society of Endocrinology [23].

The therapy was initiated with two injections at a 6-week interval and continued at intervals of 12 weeks. All the participants, Caucasian and Italian speaking, were recruited after providing informed written consent. An ethical committee reviewed the investigation protocol (nr. 1489, 10/25/2015), established in accordance with the updated version of the Declaration of Helsinki of 1964.

Subsequently, experienced psychiatrists interviewed face-to-face both the recruited patients and healthy subjects. If a psychiatric diagnosis was formulated, this was done according to the criteria of the Diagnostic and Statistical Manual of Mental Disorders, Fourth Edition (DSM-IV), and confirmed by the Structured Clinical Interview for DSM-IV, Axis I (SCID-I) and AXIS II (SCID-II) [24].

### Assessments

The experienced psychiatrists engaged were blind to whether the interviewed subjects were patients or healthy controls. They administered the validated version of the Italian Quality of Life Enjoyment and Satisfaction Questionnaire (Q-LES-Q), Symptom Checklist-90-R (SCL-90-R), Mini-mental State Examination (MMSE), Temperament and Character Inventory-Revised (TCI-R), and COPE Inventory.

#### Quality of life

The Q-LES-Q [25] is a self-report questionnaire designed to measure the degree of enjoyment and satisfaction, which proves to be better with higher scores in specific life domains that encompass eight areas: (1) physical health, (2) subjective feelings, (3) work, (4) household duties, (5) school/class work, (6) leisure time activities, (7) social relations, (8) general activities. Respondents filled in the fields concerning the areas of work, household duties, and school/class work only if they could provide such information. Items were rated on a 5-point Likert scale. We also evaluated one item of general activities to determine general life satisfaction over the before the assessment, along with two subitems (not included in the total score) regarding sexual performance and physical health satisfaction.

#### Psychopathological and cognitive evaluation

We examined psychopathology using the SCL-90-R checklist and the MMSE. The SCL-90-R is a multidimensional self-report symptom inventory designed to analyze various psychological problems and psychopathological symptoms [26]. The checklist includes 90 items based on a 5-point scale (1 = no problem to 5 = very serious) that measures the extent of the listed symptoms experienced in the last seven days. The items are divided into nine subscales: (1) somatization, (2) obsessive–compulsive, (3) interpersonal sensitivity, (4) depression, (5) anxiety, (6) hostility, (7) phobic anxiety, (8) paranoid ideation, (9) psychoticism. A high SCL-90-R score equates to great psychological distress. The SCL-90-R measures also three global indices: the Global Severity Index (GSI), which indicates the extent or depth of the individual psychiatric disturbance, the Positive Symptom Total (PST), which counts the total number of questions rated above one point, and the Positive Symptom Distress Index (PSDI), which indicates the intensity of symptoms.

The MMSE [27] is a 30-point test used to assess the severity and progression of cognitive impairment, and includes questions in different areas, grouped into seven categories, each representing a different domain or function: (1) orientation to time; (2) orientation to place; (3) registration of three words; (4) attention and calculation; (5) repetition of the three words; (6) language construction, (7) visual construction. A score of 23 or less generally indicates cognitive impairment, whose severity is classified as follows: 24–30 = no cognitive impairment; 18–23 = mild cognitive impairment; and 0–17 = severe cognitive impairment.

#### Personality traits identification

The TCI-R [28] is a 240-item self-report inventory used to denote the personality profile of interviewed subjects, using Cloninger's biopsychosocial model and a true–false response format. TCI-R is based on a 5-point-Likert format and operates with seven dimensions of personality traits based on four so-called temperaments and three so-called characters. The temperaments are related to the immediate responses of human beings to novelty seeking (NS), harm avoidance (HA), reward dependence (RD), and persistence (P). The characters are related to self-directedness (SD), cooperativeness (C), and self-transcendence (ST) [29].

#### Coping strategies

The COPE Inventory [30] comprises 15 four-item scales assessing a variety of coping strategies, divisible into two general types of coping: *problem-focused* and *emotion-focused.* The *problem-focused* coping strategies target the source of stress and aim to solve problems or to do something to alter the source of the stress. They include active-coping (taking steps to eliminate the problem), planning (thinking about dealing with the problem), suppression of competing activities (focusing only on the problem), restraint-coping (waiting for the right moment to act), and use of instrumental social support (seeking advice from others). The *emotion-focused* coping strategies, instead, try to reduce negative thoughts and feelings associated with the stressor and aim to manage the emotional distress associated with (or cued by) the situation. *Emotion-focused* coping strategies include positive reinterpretation and growth (framing the stressor in positive terms), acceptance (learning to accept the problem), denial (refusing to consider the problem as real), turning to religion (using faith for support), and emotional social support (seeking sympathy from others). The COPE Inventory, additionally, comprises other strategies such as the focus on and venting emotions (wanting to express feelings), behavioral disengagement (giving up trying to deal with the problem), mental disengagement (distracting self from thinking about the problem), substance use (using alcohol or drugs to reduce distress), and humour (making light of the problem).

Using the dispositional response format, participants indicated how frequently they employed each coping strategy on a four-point scale anchored by ‘usually do not do this at all’ and ‘usually do this a lot’.

### Measurement of total plasma testosterone

Total plasma testosterone concentrations in KS patients were measured twice: after completed one-year androgen treatment and one month after the last injection. The mean resulted from the two independent measurements, taken at a distance of one month one from the other. In healthy control subjects, androgen total plasma concentrations were measured at admission, and one month later. Blood samples were collected in the morning and sent to the analysis laboratory of the University of Campania ‘Luigi Vanvitelli’ shortly after the blood was drawn. Serum levels of total testosterone were measured using an automated immunoassay (DiaSorin Liaison^®^, Saluggia, VC, Italy), already adopted by Di Minno et al. [31]. The normal reference range for total testosterone was 6.77–31.06 nM/L.

### Statistical analysis

Continuous variables are indicated as mean ± SD. We evaluated the normality of the distribution curves by the Kolmogorov–Smirnov test and the differences by the Student’s *t* test or Mann–Whitney *U* test, where appropriate. Categorical variables are indicated as percentages. We tested for significance the differences between the KS group and the matched healthy group by an unpaired two-sided Student’s *t* test for normally distributed variables.

We analysed correlations between Q-LES-Q and the other scales—MMSE, SCL-90, TCI-R and COPE Inventory—by Spearman’s rho test. A multivariate regression model was finally applied to detect possible predictors of general and sexual life satisfaction. The model was controlled for potential confounders, such as age at onset and admission, education level, marital status, MMSE total score, medical comorbidities, and total plasma testosterone levels.

Data analysis was performed using SPSS Statistics, Version 18.0 (2009). A two-tailed *p* value ≤ 0.05 was considered statistically significant.

## Results

### Descriptive data

Sociodemographic, clinical variables, and mean total testosterone plasma concentration of KS patients and matched healthy controls are illustrated in Table 1. No patients’ family members had a history of psychiatric disorders; five patients claimed to have suffered from psychiatric disorders, namely general anxiety (*n* = 2), depression (*n* = 2), and specific phobia (*n* = 1). When compared to healthy controls, KS patients showed a lower concentration of total plasma testosterone (*p* < 0.001).

**Table 1:** Sociodemographic and clinical characteristics of the 23 enrolled patients with a verified diagnosis of Klinefelter syndrome and matched healthy controls

	Healthy controls	KS patients	*p* value
Age, years (mean ± SD)			
At admission	36.3 ± 9.6	36.2 ± 9.4	0.974
At diagnosis	–	26.5 ± 8.3	
Educational level (*n*, %)			
Elementary school	1 (4.3%)	1 (4.3%)	0.823
Middle school	8 (34.8%)	8 (34.8%)	
High school	7 (30.4%)	8 (34.8%)	
University	7 (30.4%)	6 (26.1%)	
Marital status (*n*, %)			
Single	9 (39.1%)	11 (47.8%)	0.758
Married	11 (47.8%)	9 (39.1%)	
Divorced	3 (13.0%)	3 (13.0%)	
Occupational status (*n*, %)			
Employed	18 (78.3%)	15 (65.2%)	0.454
Unemployed	5 (21.7%)	8 (34.8%)	
Stressful life events (*n*, %)			
Illness of close family member/friend	–	1 (4.3%)	
Death of close family member/friend	–	2 (8.7%)	
Stress/problems at work/school	–	6 (26.1%)	
Stress/problems at home	–	9 (39.1%)	
Sex difficulties	–	3 (13.0%)	
Smoking habit	7 (30.4%)	12 (52.2%)	0.215
Use of psychoactive drugs	–	2 (8.7%)	
Use of alcohol	–	13 (56.5%)	
Psychiatric history			
Personal	–	5 (21.7%)	
Familiar	–	0 (0%)	
Medical comorbidities			
Hypertension Diabetes mellitus type 2 Obesity Hyperlipidaemia Metabolic syndrome	1 (4.3%) – – 1 (4.3%) –	2 (8.7%) 2 (8.7%) 3 (13.0%) 4 (17.4%) 3 (13.0%)	0.549 0.343
Total testosterone plasma concentration (nM/L) (mean ± SD)	29.0 ± 1.8	3.4 ± 4.7	0.001

### QoL, psychopathological status, coping strategies and personality traits: data of study sample outcome

Q-LES-Q subscales analysis disclosed significant differences when comparing KS patients to healthy controls. The interviewed patients reported being quite dissatisfied with their physical health (*p* ≤ 0.038), social relations (*p* ≤ 0.003), and general activities (*p* ≤ 0.045), as evidenced by the lower scores reported in Table 2. The analysis of general activities subitems revealed a significant dissatisfaction of KS patients with their general life (*p* < 0.05), sexual drive and performance (*p* < 0.05), and physical health (*p* < 0.05), with leisure time activities during the last week (*p* < 0.05) being the sole source of satisfaction (Table 2).

**Table 2:** Mean total score (± SD) of Q-LES-Q subscales in healthy controls (*n* = 23) and in patients with Klinefelter syndrome (KS) (*n* = 23)

	Healthy controls	KS patients	*p* value
Q-LES-Q subscales			
Physical health/activities	60.1 ± 20.7	55.9 ± 11.5	0.038
Subjective feelings	76.0 ± 14.6	67.6 ± 23.6	0.177
Work	79.1 ± 9.9	73.7 ± 18.5	0.267
Household duties	52.9 ± 23.6	63.2 ± 29.7	0.294
School/class work	73.1 ± 15.7	73.6 ± 15.5	0.957
Leisure time activities	65.8 ± 17.8	76.1 ± 15.2	0.05
Social relations	74.0 ± 11.9	51.3 ± 17.3	0.003
General activities	61.0 ± 13.3	52.1 ± 20.3	0.045
Q-LES-Q subitems			
General life satisfaction	3.9 ± 0.8	3.2 ± 1.2	0.05
Sexual performance satisfaction	4.0 ± 0.6	2.8 ± 1.3	0.05
Physical health satisfaction	3.4 ± 0.9	2.9 ± 1.2	0.05

The analysis of SCL-90-R evidenced that KS patients when compared to healthy subjects, reported higher scores on most subitems indicating psychological distress. Severe symptoms were obsessions and compulsions (*p* < 0.008), hanger-hostility (*p* < 0.028), phobias (*p* < *0.038*), and psychoticism (*p* < 0.013), although a trend of higher scores emerged for some of the remaining SCL-90-R subscales (Table 3). GSI indeed displayed a significantly higher severity of distress mean score in KS patients when compared to healthy controls (*p* < 0.030) (Table 3), as also occurred with the PSDI score (*p* < 0.004), which indicated the intensity due to symptoms.

**Table 3:** Mean total score (± SD) of SCL-90 subscales and MMSE in matched healthy controls (*n* = 23) and patients with Klinefelter syndrome (KS) (*n* = 23)

	Healthy controls	KS patients	*p* value
SCL-90 subscales			
Somatization	0.92 ± 0.48	1.06 ± 0.88	0.086
Obsessive–compulsive	0.55 ± 0.60	1.16 ± 0.79	0.008
Interpersonal sensitivity	0.98 ± 0.84	0.82 ± 0.68	0.437
Depression	0.69 ± 0.62	0.91 ± 0.71	0.070
Anxiety	1.02 ± 0.58	1.00 ± 0.81	0.108
Anger-hostility	0.39 ± 0.45	0.89 ± 0.89	0.028
Phobias	0.07 ± 0.21	0.32 ± 0.48	0.038
Paranoid ideation	0.57 ± 0.62	0.94 ± 0.91	0.099
Psychoticism	0.24 ± 0.37	0.59 ± 0.50	0.013
PST	26.7 ± 21.7	38.1 ± 23.0	0.107
PSDI	1.3 ± 0.5	1.8 ± 0.5	0.004
GSI	0.5 ± 0.5	0.9 ± 0.6	0.030
MMSE	29.6 ± 0.9	26.9 ± 2.4	0.0001

Interpersonal sensitivity (*B* = − 8.42; *β* = − 2.64; *p* < 0.005), somatization (*B* = − 2.46; *β* = − 0.82; *p* < 0.02), anxiety (*B* = − 4.27; *β* = − 1.93*; p* < 0.03), depression (*B* = − 0.76; *β* = − 0.55; *p* < 0.02), and psychoticism (*B* = − 0.47; *β* = − 0.47; *p* < 0.03) SCL-90-R subscales negatively influenced the physical health, social relations and general activities areas of Q-LES-Q. In addition, all three indices of SCL-90-R (PST, PSDI, and GSI) negatively correlated with physical health, social relations, and general activities areas of Q-LES-Q (*p* < 0.05).

The mean total score of MMSE was significantly different in KS patients compared to healthy controls (*p* < 0.0001) (Table 3). Specifically, 16 patients showed a normal range, and only five a mild-borderline score range. Correlation analysis evidenced that the MMSE score was positively associated with the interpersonal sensitivity subscale of SCL-90-R (*p* < 0.04). Also, the MMSE score positively influenced the subjective feelings area of Q-LES-Q (*B* = 6.98, *β* = 0.63, *p* < 0.03).

COPE Inventory also evidenced that KS patients, when compared to healthy subjects, used mainly *emotion-focused* coping strategies to manage emotional distress such as denial (*p* < 0.007) and turning to religion (*p* < 0.03). Other remarkable coping strategies that emerged were focused on and venting of emotions (*p* < 0.01) and behavioral disengagement (*p* < 0.03) (Table 4). Correlation analysis between Q-LES-Q areas and COPE Inventory subscales turned out to be positive for most strategies. *Active coping* positively influenced physical health (*B* = 5.96; *β* = 0.72; *p* < 0.003) and social relations (*B* = 3.16; *β* = 0.56; *p* < 0.03). Moreover, a significant but negative correlation was evidenced between plasma testosterone levels and mental disengagement (*B* = − 45.3; *β* = − 0.98; *p* < 0.001).

**Table 4:** Mean total score (± SD) of COPE Inventory subscales in healthy controls (*n* = 23) and in patients with Klinefelter syndrome (KS) (*n* = 23)

COPE inventory subscales	Healthy controls	KS patients	*p* value
Problem-focused			
Active coping	11.8 ± 1.6	12.1 ± 2.9	0.649
Planning	12.6 ± 1.7	12.1 ± 3.1	0.543
Suppression of competing activities	10.2 ± 2.3	9.9 ± 2.8	0.632
Restraint coping	10.1 ± 2.1	10.1 ± 2.3	0.945
Use of instrumental social support	11.2 ± 2.6	11.2 ± 2.5	0.952
Emotion-focused			
Positive reinterpretation and growth	11.5 ± 2.2	12.7 ± 2.2	0.074
Acceptance	10.8 ± 2.3	10.3 ± 2.8	0.552
Denial	5.3 ± 1.7	7.4 ± 3.0	0.007
Turning to religion	6.1 ± 3.5	8.8 ± 4.2	0.033
Use of emotional social support	10.3 ± 2.9	10.4 ± 3.2	0.959
Other included coping strategies			
Focus on and venting of emotions	8.1 ± 2.8	10.2 ± 2.6	0.019
Behavioral disengagement	6.1 ± 1.5	7.7 ± 2.9	0.032
Mental disengagement	7.7 ± 2.2	8.7 ± 2.8	0.210
Substance use	4.3 ± 0.9	4.9 ± 2.7	0.397
Humor	7.5 ± 2.9	7.9 ± 3.2	0.730

The analysis of TCI-R temperament dimensions evidenced no noticeable difference when we compared KS patients to healthy controls. Among character dimensions, instead, self-directedness (*p* < 0.05) and self-transcendence (*p* < 0.0001) were significantly higher in androgen-treated KS patients than in healthy controls, as shown in Table 5. Correlations between physical health, social relations, and general activities areas of Q-LES-Q and TCI subscales were significant at *p* < 0.05 and positive, except for HA, which was negatively correlated. *Cooperativeness* subscale of TCI-R, in particular, represented a character trait influencing the subjective feelings (*B* = 0.99; *β* = 0.56; *p* < 0.03) and social relations areas (*B* = 0.64; *β* = 0.60; *p* < 0.02) of QoL.

**Table 5:** Mean total score (± SD) of TCI-R subscales in healthy controls (*n* = 23) and in patients with Klinefelter syndrome (KS) (*n* = 23)

TCI-R subscales	Healthy controls	KS patients	*p* value
Novelty seeking (NS)	106.8 ± 10.7	107.1 ± 16.5	0.938
Harm avoidance (HA)	94.4 ± 15.7	93.0 ± 21.0	0.811
Reward dependence (RD)	102.9 ± 10.1	99.1 ± 14.3	0.318
Persistence (P)	111.9 ± 15.9	117.9 ± 20.5	0.300
Self-directedness (SD)	149.9 ± 20.5	135.3 ± 26.2	0.05
Cooperativeness (C)	130.4 ± 10.1	125.2 ± 13.4	0.159
Self-transcendence (ST)	56.6 ± 13.4	77.2 ± 18.2	0.0001

### Predictive factors of satisfaction in KS patients

Multivariate regression analysis, when adjusted for age at onset and study entry, MMSE total score, medical comorbidities, and total plasma testosterone levels, evidenced that only some subscales of SCL-90-R proved to be predictors of general and sexual life satisfaction (Table 6). In particular, SCL-90-R subscales scores for somatization (*p* < 0.017) and depression (*p* < 0.019) turned to be predictors of general life dissatisfaction. On the other hand, depression (*p* < 0.009), anger-hostility (*p* < 0.009), and paranoid ideation (*p* < 0.029) subscales scores emerged as predictors of sexual life dissatisfaction, while interpersonal sensitivity proved to be a positive predictor (*p* < 0.004).

**Table 6:** Predictors of general and sexual life satisfaction in KS patients by applying a multivariate regression model

Variables	General life satisfaction			Sexual life satisfaction		
	OR	CI 95%	*P*	OR	CI 95%	*p*
SCL-90-R subscales						
Somatization	− 0.772	− 1.395/− 0.150	0.017	− 0.501	− 1.117/0.114	0.073
Obsessive–compulsive	− 0.185	− 1.108/0.739	0.310	− 0.422	− 1.761/0.917	0.128
Interpersonal sensitivity	0.420	− 0.115/0.955	0.992	0.742	− 0.258/1.743	0.004
Depression	− 1.027	− 1.876/− 0.179	0.019	− 1.799	− 3.016/− 0.581	0.009
Anxiety	0.767	− 0.076/1.611	0.095	− 0.243	− 1.858/1.371	0.689
Anger-hostility	0.533	− 0.280/1.347	0.052	− 1.363	0.451/2.275	0.009
Phobias	0.692	− 0.487/1.871	0.079	0.211	− 1.406/1.829	0.349
Paranoid ideation	− 0.480	− 1.130/0.170	0.793	− 0.894	− 1.668/− 0.120	0.029
Psychoticism	− 0.411	− 1.793/0.971	0.309	0.848	− 1.023/2.719	0.395

The model was adjusted for age at onset and at admission, MMSE total score, medical comorbidities, and total testosterone plasma levels

## Discussion

The present study is the first to report that KS patients, treated for one year with testosterone undecanoate, do not yield a QoL compared to matched healthy controls. Besides, our KS patients showed self-directedness and self**-**transcendence and used prevalent *emotion-focused* coping strategies to reduce distress. We finally reported that in such patients, depression and somatization might be crucial predictors of general life dissatisfaction, while anger-hostility, paranoid ideation, and depression of sexual life dissatisfaction.

Low levels of testosterone can compromise sexual function, energy levels, body composition, and cognitive function in KS patients, whose QoL may deteriorate [32]. Testosterone replacement therapy outcomes observed in KS patients are similar to those reported in males with hypogonadism unrelated to KS [15]. In particular, body composition and bone mineral density appear better in treated than untreated hypogonadal KS patients [18].

Finas et al. [19] reported a reduced physical health index of QoL associated with no changes in mental health index in hypogonadal patients when compared to matched aged controls. However, psychopathological dimensions were not analyzed. Also, Meikle et al. [33] reported that the use of transdermal testosterone patches improved sexual function, increased libido, and decreased fatigue in KS patients, but did not address the degree of satisfaction and quality of life. Our KS patients showed lower scores on physical, social, and psychological scales of QoL when compared to matched healthy subjects, in line with the results of de Ronde et al. [34]. Leisure time activities proved to be the only remarkable source of satisfaction. Indeed, the analysis of a few items of general activity subscale of Q-LES-Q, displayed a general life, physical health, and sexual performance dissatisfaction in our patients.

Limited data are available about the effects of testosterone replacement on cognitive function. Nonetheless, a few studies reported that androgen replacement therapy showed a variable impact on cognitive functions [35–38] though producing positive effects on several aspects of psychosocial function. Most of our KS testosterone-treated patients showed an MMSE score within the norm, even though with remarkable differences when compared to that of healthy controls. However, it is still unclear whether testosterone treatment may have an impact on cognition, as suggested by a part of studies that report moderate beneficial effects [39, 40], or no improvement [38, 41, 42]. Genetic factors, as well, may manifest QoL through characteristic cognitive and language deficits and, secondarily, lead to adaptation and behavioral problems during adolescence [7]. Impaired measures of verbal skills, high incidence of dyslexia, and social dysfunctions are among the most consistently reported behavioral phenotypes. KS patients may primarily manifest difficulties in social adjustment and in coping with social situations. Indeed, high levels of distress during social interactions, are consistent with reports of social anxiety and withdrawal, shyness, and greater perceived stigmatization [8].

Our data on the influence of psychological distress on the QoL of androgen-treated KS patients are generally in line with those of the current literature [3, 8, 9, 11, 38]. In our study, KS patients experienced more obsessive, intrusive and persistent thoughts, phobic and avoidant behaviors in coping with external stimuli considered threatening (phobic anxiety), and accordingly reported more frequent hostile thoughts and behaviors (anger-hostility). They also reported an introverted and isolated lifestyle of schizoid type (psychoticism), possibly expandable to paranoid ideation and anger-hostility. Both elements result in predictors of sexual life dissatisfaction in our multivariate regression analysis, as already described on KS patients [43, 44]. Such patients may experience more anxiety and depressive symptoms compared to healthy control subjects, as reported in the literature [45, 46]. Indeed, we evidenced that depressive symptoms not only negatively influenced QoL but also represented predictors of both general and sexual life dissatisfaction. We also evidenced that interpersonal sensitivity negatively influenced the physical health, social relations, and general activities areas of Q-LES-Q, besides being a positive predictor of sexual life satisfaction. These factors may contribute to worsening the perceived disease-related stigma, which we did not measure in our KS patients [8]. Somatization proved to be a sole predictor of general life dissatisfaction, as our regression analysis displayed.

Cloninger [47] stated that psychological wellbeing and related physical health depends on the development of the three character dimensions facets, such as those originating from self-directedness (autonomy and life purpose), cooperativeness (positive relations with others), and self-transcendence (personal growth and self-actualization). The mean lower score of SD reported by our KS patients when compared to healthy subjects might be therefore considered as a latent dissatisfaction with physical, emotional, social, and sexual functioning wellbeing. The higher score of the ST subscale of TCI-R may yet delineate our patients’ attempt to foster spirituality and wellbeing through universal meanings and purposes, pursuing an utter QoL. Such an aim is also revealed by the higher score of KS patients on turning to religion subscale of COPE Inventory when compared to healthy subjects.

Emotional release is pursued through different activities, such as daydreaming, escaping through sleep, or immersing in TV to handle stressor-related thoughts and feelings.

Such activities were more intense in our cohort of KS patients compared to healthy controls, as confirmed by the high increase of the score for leisure time activities subscale of Q-LES-Q. Differently, Turriff et al. [8] observed that the use of *problem-focused* coping strategies in KS patients was positively correlated with the adaptation process, which gives insight into how effectively a person manages the stress of a health condition or health threat [48]. Furthermore, the authors reported that *emotion-focused* coping is used, instead, when patients consider stressors as something to endure. In this regard, denial and behavioral disengagement may represent two successful strategies to ensure the adaptation process of acknowledging the implications of a health threat and the outcomes of the related process [48].

For the first time in the literature, we performed a quantitative and qualitative analysis of psychopathology, temperament, and character traits of personality, and coping strategies, using a patient-centered approach instead of a disease-centered one. Indeed, our study focused on adult KS patients after a one-year standardized treatment—fixed dose of injectable testosterone undecanoate. Full compliance was achieved since the injections were administered in our outpatient unit. No surveys or cross-sectional design studies exist on patients who were drug-free and/or treated with different formulations of the androgen, and on standardized dosage or treatment duration. Furthermore, no information was provided on adherence to treatment, which may have biased the results [12, 34, 45, 49]. The mean age and educational level of our matched healthy group were not different from those of testosterone-treated KS patients, differently from what occurred in the study of de Ronde et al. [34].

Finally, we need to mention limitations pertinent to our study, such as the relatively limited number of the enrolled participants, the heterogeneity of KS concerning neuropsychological phenotype, the temporal frame (namely the week before administering the tests), and the patient’s self-assessment of general psychopathology, subject to personal perceptual bias. The psychiatric evaluation we performed by direct interviews remains the distinctive element of our study. Furthermore, the cross-sectional design is to be considered, since we analyzed data only at a specific time-point, namely at the end of one year of injectable testosterone therapy. A prospective study evaluating the effects of the androgen treatment on the psychological variables would have probably provided additional information. Moreover, in both controls and KS patients, we did not use the free-testosterone index (testosterone/sex hormone-binding globulin [SHBG]), mainly adopted to confirm the clinical diagnosis of hypogonadism or to modulate the androgen treatment [15].

Finally, our study results can be subject to recall bias for a part of clinical data collected retrospectively.

In conclusion, our study suggests that both physical and mental wellbeing are crucial for the self-perception of a satisfactory QoL. Further studies on the relationship between psychopathology, stigma, satisfaction, and body image could improve the understanding of the relevance of body image as a transdiagnostic factor, and its potential value as a target for therapies.

As stated in the constitution of WHO, “Health is a state of complete physical, mental and social wellbeing and not merely the absence of disease or infirmity” [50]. Such a definition fully adapts to patients diagnosed with KS, who suffer from a wide variety of psychophysical conditions that may considerably invalidate their lives.

On the grounds of our findings, we conclude that clinicians should consider psychopathology as a factor that may substantially influence the quality of life of patients affected by Klinefelter syndrome.

Therefore, we suggest that endocrinologists cooperate with mental health specialists to expand the field of therapeutic intervention and achieve a more positive outcome of the disease.
